# Periprosthetic fracture caused by stress shielding after implantation of a femoral condyle endoprosthesis in a transfemoral amputee—a case report

**DOI:** 10.3109/17453674.2010.533937

**Published:** 2010-11-26

**Authors:** Tina S Wik, Olav A Foss, Steinar Havik, Leif Persen, Arild Aamodt, Eivind Witsø

**Affiliations:** ^1^Department of Orthopaedic Surgery, Trondheim University Hospital; ^2^Department of Neuroscience, Norwegian University of Science and Technology (NTNU), Trondheim, Norway

A femoral condyle endoprosthesis (FCE) was implanted in a 48-year-old transfemorally amputated woman with the intention of making the amputation stump fully endbearing (Figure 1). The implant was a customized endoprosthesis of titanium alloy (Scandinavian Customized Prosthesis AS, Trondheim, Norway), based on experience of the Unique Customized Femoral Stem (Aamodt et al. 1999). Cross-sectional CT images were used to retrieve the inner cortical contours of the femoral diaphysis, and the stem was designed to fit closely within the femoral canal (Aamodt et al. 1999). The stem was fully coated with a dual layer of titanium and hydroxyapatite. During implantation, a small fissure occurred at the anterior aspect of the distal part of the femur, which was secured with 2 cerclage wires. There were no other peroperative or postoperative complications. After 6 weeks of unloading, the patient received a new artificial limb with a prosthetic socket that allowed endbearing. At the 12-month follow-up, the patient was using a knee disarticulation socket that terminated below the groin and the tuber ischiadicum. Radiographs showed improved alignment of the amputated leg (Figure 2) and the patient reported only minor stump pain, even with full endbearing. The skin was normal, probably because of the large bearing surface of the artificial condyle (Jensen 1996).

**Figure 1.**
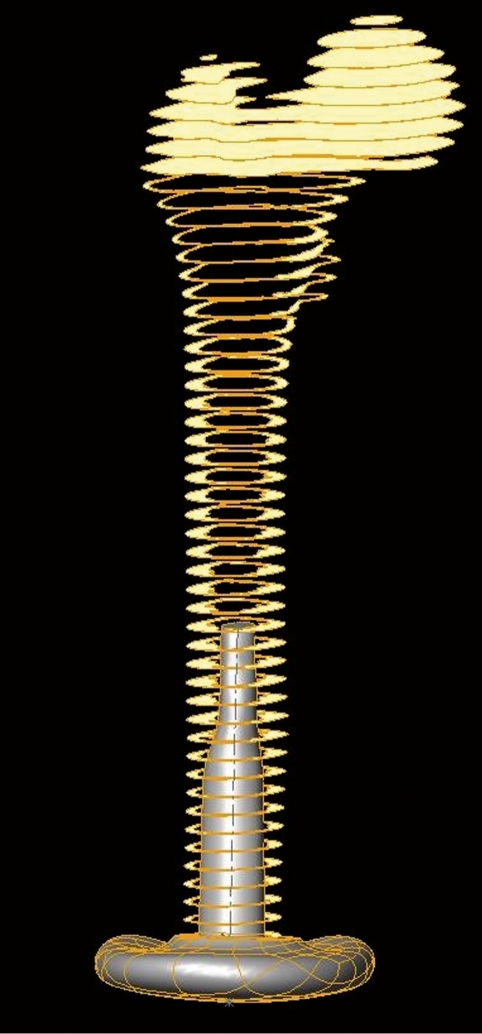
Preoperative computer construction of the femoral condyle endoprosthesis (FCE) inside the residual femur.

**Figure 2.**
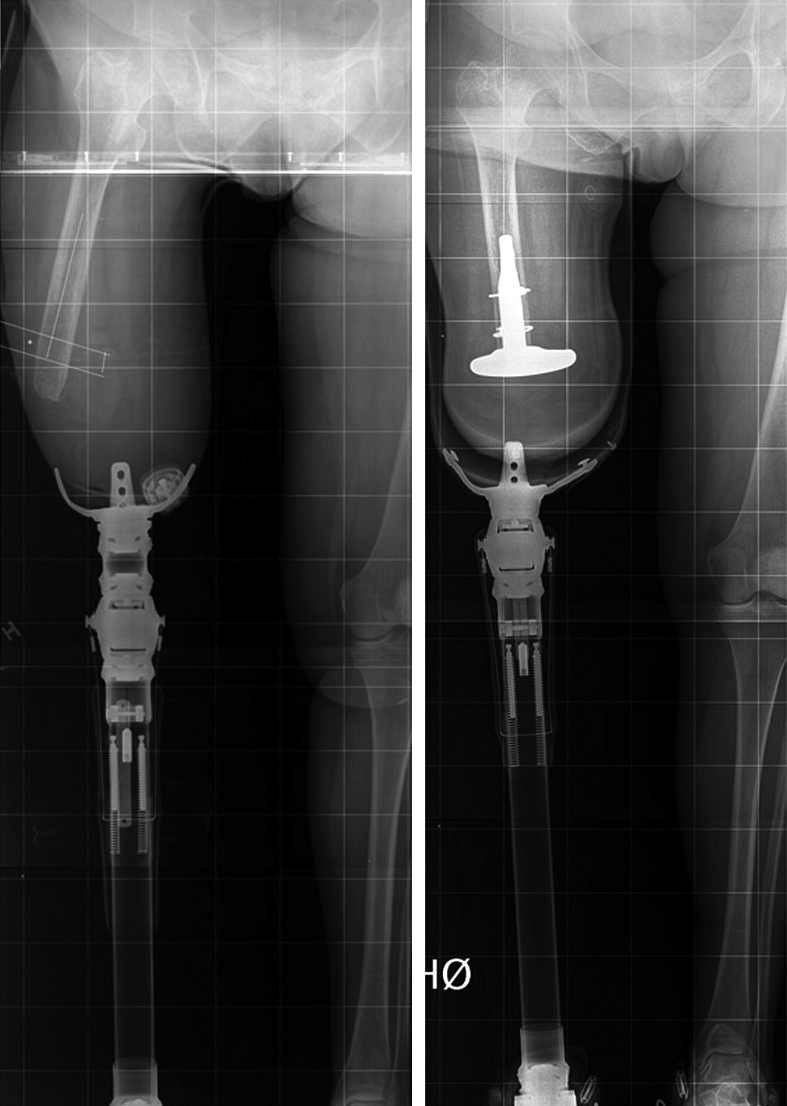
Femoral alignment before and after insertion of the FCE.

The patient experienced a minor trauma 24 months after surgery while using the external prosthesis, and radiographs revealed a periprosthetic fracture of the femur. During removal of the stem, considerable periprosthetic bone loss was found at the distal part of the stem. This bone loss had developed gradually, and could be observed on radiographs as early as 6 months after implantation of the FCE (Figure 3). Culture of tissue samples harvested during the reoperation gave no evidence of infection that could explain the bone loss. Radiographs taken 15 months after removal of the FCE showed bone apposition in the distal part of the femur (Figure 3).

**Figure 3.**
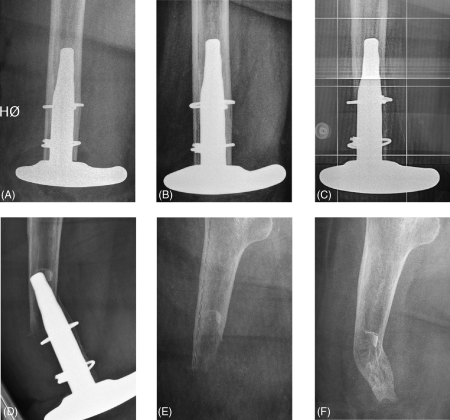
Gradual bone loss at 6 and 12 months, fracture at 24 months, and bone regeneration after removal of the implant. A. Postoperatively. B. At the 6-month follow-up. C. At the 12-month follow-up. D. Fracture at 24 months. E. Fracture postoperatively. F. 15 months after fracture.

## Discussion

Dynamic strain induced by functional loading is the controlling stimulus of bone remodeling (Ehrlich and Lanyon 2002, Lanyon 2008). Strain protection of the periprosthetic bone occurs when the load is transferred through the implant, and hence bypasses the bone (Prendergast 2001, McPherson 2004). The periprosthetic bone resorption observed is commonly referred to as stress shielding.

Stress shielding is well known and extensively documented around the femoral stem in total hip arthroplasties (THAs) (Prendergast 2001, McPherson 2004, Glassman et al. 2006). It is also described in total knee replacement, tumor prostheses (Lan et al. 2000, Li and Nilsson 2000, van Loon et al. 2001), and osseointegrated implants (Xu and Robinson 2008). Even though stress shielding is commonly observed after insertion of cementless femoral THA stems, clinically adverse effects due to this phenomenon have not been observed (Engh et al. 2003, 2009, Glassman et al. 2006).

Our patient experienced a clinical failure caused by severe bone resorption, probably due to stress shielding. The stiffness of the implant material and the femur are one of the main determinants in bone remodeling around an implant (Bobyn et al. 1992, Engh et al. 1999, Glassman et al. 2006). The stiffness of the stem is a function of both the material modulus and the implant geometry. The stem diameter is, however, of considerably more importance for the stiffness of the implant than the material in itself (Bobyn et al. 1992). The FCE was an uncemented press-fit implant, and therefore the diameter of the stem was quite wide. The dimension of the FCE was based on CT scans of the femur, identifying the inner cortical contours of the distal femur. A previous study showed that bone tissue with a density of approximately 600 Hounsfield units on CT scans can provide sufficient mechanical stability to an implant (Aamodt et al. 1999). In our patient, the residual femur must be expected to be osteoporotic 10 years after the amputation (Sherk et al. 2008) and a design criterion based on density could thus yield an even wider stem diameter.

Furthermore, the extent of porous coating has been shown to influence periprosthetic bone loss, as it appears that bone resorption occurs in the areas where there is ingrowth of bone into the implant. Extensively coated femoral stems, such as the FCE, will have a more pronounced bone loss than proximally or partially coated stems (Bobyn et al. 1992, Blunn et al. 2000, Yamaguchi et al. 2000, Werner et al. 2005).

The expected osteoporosis of the residual femur in our patient preoperatively would probably add further to the risk of periprosthetic bone resorption; both systemic and local low bone mineral density aggravates periprosthetic bone resorption after THA (Venesmaa et al. 2003, Rahmy et al. 2004, Alm et al. 2009).

To our knowledge, the same degree of dramatic bone loss as observed for the FCE has not been observed in bulky femoral THA stems. One reason for this could be the influence of muscle loading. The muscle insertions into the greater and the lesser trochanter will give loading of the proximal femur, while the distal part of the femur in a transfemoral amputee will not have a normal muscle loading.

Of course, we cannot rule out the possibility that factors other than stress shielding contributed to the extensive bone loss around the FCE stem. The intraoperative fissure made cerclage fixation necessary, and therefore soft tissue was stripped off the femur distal to the most distal wire. This could have contributed to devascularization of the bone. Furthermore, unknown and individual factors may have contributed to bone loss.

It seems rather obvious that the extensive bone resorption was one of the main causes of the periprosthetic fracture in our case. Although a loose stem could have contributed to the fracture, it is unlikely that the massive bone loss was caused by instability of the stem. We have no reason to suspect that the femoral stem was loose before the periprosthetic fracture, based on the perioperative judgement of the operating surgeons and the patient's clinical performance—with less and less pain in the amputation stump and the thigh, even during full weight bearing. Furthermore, the distal medial part of the FCE stem had a small defect of the surface coating. A corresponding artefact in the residual femur could be seen on the radiographs from the time of fracture and after removal of the FCE (Figure 3).

In retrospect, the risk factors associated with stress shielding are consistent with the high degree of bone loss observed in this patient. After removal of the implant, radiographs showed evidence of bone regeneration in the distal femur. This may have been caused by removal of the stress-bypassing component, and the re-introduction of some axial load to the distal femur.

This is a unique patient case with failure probably caused by extreme stress shielding after implantation of an experimental implant. This should also be a warning when developing new implants, as it shows that stress shielding can actually have serious consequences if the bone loss is severe enough.
